# Trends and outcomes of heart failure hospitalizations during COVID-19 pandemic

**DOI:** 10.1186/s12889-025-21995-y

**Published:** 2025-03-04

**Authors:** Muni Rubens, Venkataraghavan Ramamoorthy, Anshul Saxena, Atulya Aman Khosla, Mayur Doke, Peter McGranaghan, Sandeep Appunni, Yanjia Zhang, Daniel Körfer, Sandra Chaparro, Javier Jimenez

**Affiliations:** 1 Miami Cancer Institute, Baptist Health South Florida, Miami, FL USA; 2 Herbert Wertheim College of Medicine, Florida International University, Miami, FL USA; 3 Universidad Espíritu Santo, Samborondón, Ecuador; 4 Center for Advanced Analytics, Baptist Health South Florida, Miami, FL USA; 5 William Beaumont University Hospital, Royal Oak, MI 48073 USA; 6 University of Miami, Miami, FL USA; 7 Semmelweis Doctoral College, Semmelweis University, Budapest, Hungary; 8 Government Medical College, Kozhikode, Kerala India; 9 Miami Cardiac & Vascular Institute, Baptist Health South Florida, Miami, FL USA

**Keywords:** COVID-19, Heart failure, In-hospital mortality, Mechanical ventilation, Mechanical circulatory support, Vasopressor use, Acute respiratory distress syndrome

## Abstract

**Background:**

COVID-19 has affected many hospitalizations. In this study, we intended to understand the effects of COVID-19 pandemic on heart failure hospitalizations in the state of California.

**Method:**

This study was a retrospective analysis of California State Inpatient Database during March to December of 2019 and 2020. Adult hospitalizations with heart failure were included for the analysis. Main outcome variables were in-hospital mortality, mechanical ventilation, mechanical circulatory support, vasopressor use, and acute respiratory distress syndrome (ARDS).

**Results:**

There were 450,771 (53.7%) heart failure hospitalizations during March to December of 2019, compared to 388,795 (46.3%) during March to December of 2020 (relative decrease, 13.7%). Heart failure hospitalization rates were lower during 2020, compared to 2019. Comparison of adverse hospital outcomes across the two-time frames showed that in-hospital mortality (2.9% versus 2.7%, *P* = 0.003), mechanical circulatory support (0.7% versus 0.5%. *P* < 0.001), vasopressor use (1.3% versus 1.0%, *P* < 0.001), and ARDS (0.1% versus 0.06%, *P* = 0.007) were significantly higher among hospitalizations in 2020. Regression analysis showed that the odds of in-hospital mortality (OR, 1.09; 95% CI, 1.06–1.11), mechanical ventilation (OR, 1.07; 95% CI, 1.05–1.09), vasopressor use (OR, 1.07; 95% CI, 1.04–1.10), and ARDS (OR, 1.74; 95% CI, 1.58–1.91) were significantly higher among heart failure hospitalizations in 2020.

**Conclusions:**

Our study found that patients with heart failure hospitalized during the COVID-19 pandemic had greater in-hospital adverse events such as greater in-hospital mortality, mechanical ventilation use, vasopressor use, and ARDS. These findings warrant that heart failure required prompt hospitalization and treatment irrespective of restrictive mandates during COVID-19 pandemic.

**Supplementary Information:**

The online version contains supplementary material available at 10.1186/s12889-025-21995-y.

## Introduction

The Novel Coronavirus Disease 2019 (COVID-19) has been one of the greatest global public health challenges in the recent era. Many countries have struggled to control the transmission of the disease in spite of various public health measure such as lockdowns, quarantines, mask mandates, and travel restrictions [1]. This was also accompanied with significant decreases in hospitalizations for conditions other than COVID-19. Though many minor ailments were successfully managed through novel methods such as telemedicine care, major conditions such as heart failure have been adversely affected due to prioritization of hospitals and resources for COVID-19 [2]. This is concerning because heart failure is a serious condition that requires hospitalizations, investigations, and prompt management. The current prevalence of heart failure among US population is nearly 6 million, many of whom require hospitalizations for its management [3]. Studies from Europe and the UK have shown that COVID-19 pandemic has significantly impacted hospitalizations for heart failure [4, 5]. Similar results have also been reported in US states such as Tennessee, Philadelphia, and Mississippi [6–8]. However, similar studies on the effects of COVID-19 on heart failure hospitalizations are lacking in the states situated in the west coast of US. In this study, we intended to understand these effects on heart failure hospitalizations using data from a largescale repository such as State Inpatient Database (SID) for the state of California which has recorded the greatest number of COVID-19 cases within the US [9].

## Methods

### Study design and data source

We conducted a retrospective analysis of data from California State Inpatient Database (SID) collected during 2019 and 2020. The Agency for Healthcare Research and Quality (AHRQ) developed the SID database to collect statewide inpatient clinical data from patients admitted to participating hospitals within the state [10]. The SID annually collects discharge data from more than 90% of patients admitted to community hospitals. The Strengthening the Reporting of Observational Studies in Epidemiology (STROBE) guideline was followed for ensuring the quality of our study [11].

### Study population

Data from adult (≥ 18 years of age) heart failure hospitalizations that occurred during March to December of 2019 and March to December of 2020 were included for the analysis. We used the International Classification of Diseases, Tenth Revision, Clinical Modification (ICD-10-CM) diagnosis and procedure codes for identifying hospitalizations and procedures (Supplementary Table 1).

### Study variables and outcomes

The primary outcome of the study were trends in heart failure hospitalizations and in-hospital mortality, and secondary outcomes were mechanical ventilation, mechanical circulatory support, vasopressor use, and acute respiratory distress syndrome (ARDS). Independent variables included age, sex, race, insurance status, clinical risk profile, and Elixhauser comorbidity index. ICD-10-CM diagnosis and procedure codes were used for identifying these variables (Supplementary Table 1).

The study was reviewed by the Florida International University’s Institutional Review Board, which exempted the study from institutional review board approval and waived the requirement for informed consent because it uses previously collected deidentified data stored in SID. This study was performed in accordance with the Declaration of Helsinki.

### Statistical analysis

We used descriptive statistics to understand the differences in demographic and clinical characteristics between heart failure hospitalizations that occurred during March to December of 2019 versus March to December of 2020. Frequencies and percentages were used to describe categorical variables while median and interquartile range were used for continuous variables. We used Rao-Scott chi-square test to compare categorical variables, and t test and Mann-Whitney U test for continuous variables. Monthly trends in heart failure and COVID-19 hospitalizations were graphically tabulated and plotted against one another. We conducted propensity score matching (PSM) to account and control for potential differences in demographics and clinical profiles between hospitalizations that occurred during the two-time frames. For PSM. we used a 1:1 greedy matching algorithm with a caliper size of 0.25. We used density plots for comparing matching to ensure adequate match between the two groups. We used conditional logistic regressions to find differences in strength of association between hospitalization during 2019 and 2020 with regards to outcome variables such as in-hospital mortality, mechanical ventilation, mechanical circulatory support, vasopressor use, and ARDS. In our models, we controlled for covariates such as age, sex, race, insurance status, and clinical risk profiles such as hypertension, diabetes mellitus, hyperlipidemia, obesity, atrial fibrillation, coagulation disorder, peripheral vascular disease, liver disease, renal failure, prior myocardial infarction (MI), prior percutaneous coronary intervention (PCI), prior coronary artery bypass graft (CABG), tobacco use, alcohol abuse, drug abuse, cardiac arrest, and cardiogenic shock. Statistical significance was set at *P* < 0.05 and all tests were two sided. All statistical analyses were conducted using SAS, version 9.4 (SAS Inc., Cary, NC).

## Results

There were 101,032 (56.0%) heart failure hospitalizations during March to December of 2019, compared to 79,637 (44.0%) during March to December of 2020 (relative decrease, 21.2%). Hospitalizations for COVID-19 increased from 2,252 to 46,217 during the same period (relative increase, 19521.3%). During both periods, nearly 45% of the patients were in the age group 65–85 years. There were greater number of males during both time frames. Majority of the patients were Whites, followed by Hispanics, Blacks, and Asian or Pacific Islander and Native American. The majority of the patients had Medicare coverage, followed by Medicaid and private insurance. All demographic characteristics differed significantly between the two-time frames. Comparison of clinical risk profiles showed that the rates of hypertension, prior MI, prior PCI, and prior CABG were significantly higher among heart failure hospitalizations in 2019, whereas obesity, atrial fibrillation, coagulation disorder, liver disease, tobacco use, alcohol abuse, drug abuse, cardiac arrest, and cardiogenic shock were significantly higher among heart failure hospitalizations in 2020. Elixhauser comorbidity index scores ≥ 3 was observed among 69.4% of heart failure hospitalizations in 2019 compared to 70.1% in 2020. Table 1 shows the demographic and clinical risk profile of heart failure hospitalizations during March to December of 2019 and 2020.

**Table 1:** Demographic and clinical characteristics of heart failure hospitalizations during 2019 and 2020

Characteristic	2019 March to December *n* = 101,032 (56.0%)	2020 March to December *n* = 79,637 (44.0%)	*P* value
Age, *n* (%)			< 0.001
18–44 years	6333 (6.3%)	5586 (7.0%)	
45–64 years	30,016 (29.7%)	25,041 (31.4%)	
65–85 years	45,878 (45.4%)	36,292 (45.6%)	
≥ 86 years	18,805 (18.6%)	12,718 (16.0%)	
Sex, *n* (%)			< 0.001
Male	56,147 (55.6%)	45,658 (57.3%)	
Female	44,881 (44.4%)	33,973 (42.7%)	
Race/ethnicity, *n* (%)			< 0.001
White	46,453 (46.3%)	36,461 (46.2%)	
Black	14,756 (14.7%)	12,059 (15.3%)	
Hispanic	25,016 (24.9%)	19,543 (24.7%)	
Asian or Pacific Islander and Native American	9744 (9.7%)	6926 (8.8%)	
Other	4441 (4.4%)	3981 (5.0%)	
Insurance, *n* (%)			< 0.001
Medicare	65,340 (64.7%)	49,767 (62.5%)	
Medicaid	23,716 (23.5%)	20,363 (25.6%)	
Private insurance	9164 (9.1%)	7271 (9.1%)	
Uninsured	1287 (1.3%)	983 (1.2%)	
Other	1511 (1.5%)	1232 (1.5%)	
Clinical risk profile, *n* (%)			
Hypertension	92,766 (91.8%)	72,876 (91.5%)	0.018
Diabetes mellitus	10,558 (10.5%)	8221 (10.3%)	0.379
Hyperlipidemia	54,410 (53.9%)	42,581 (53.5%)	0.102
Obesity	25,186 (24.9%)	21,429 (26.9%)	< 0.001
Atrial fibrillation	31,054 (30.7%)	27,236 (34.2%)	< 0.001
Coagulation disorder	9504 (9.4%)	8242 (10.3%)	< 0.001
Peripheral vascular disease	21,701 (21.5%)	16,978 (21.3%)	0.410
Liver disease	7098 (7.0%)	6478 (8.1%)	< 0.001
Renal failure	55,712 (55.1%)	43,658 (54.8%)	0.172
Prior MI	15,959 (15.8%)	12,047 (15.1%)	< 0.001
Prior PCI	10,988 (10.9%)	8085 (10.2%)	< 0.001
Prior CABG	11,287 (11.2%)	7830 (9.8%)	< 0.001
Tobacco use	15,244 (15.1%)	12,700 (15.9%)	< 0.001
Alcohol abuse	5330 (5.3%)	4778 (6.0%)	< 0.001
Drug abuse	12,779 (12.6%)	12,120 (15.2%)	< 0.001
Cardiac arrest	677 (0.7%)	775 (1.0%)	< 0.001
Cardiogenic shock	2836 (2.8%)	2794 (3.5%)	< 0.001
Elixhauser comorbidity index, *n* (%)			< 0.001
0	3282 (3.2%)	2374 (3.0%)	
1 or 2	27,672 (27.4%)	21,419 (26.9%)	
≥ 3	70,078 (69.4%)	55,844 (70.1%)	

**Fig. 1:**
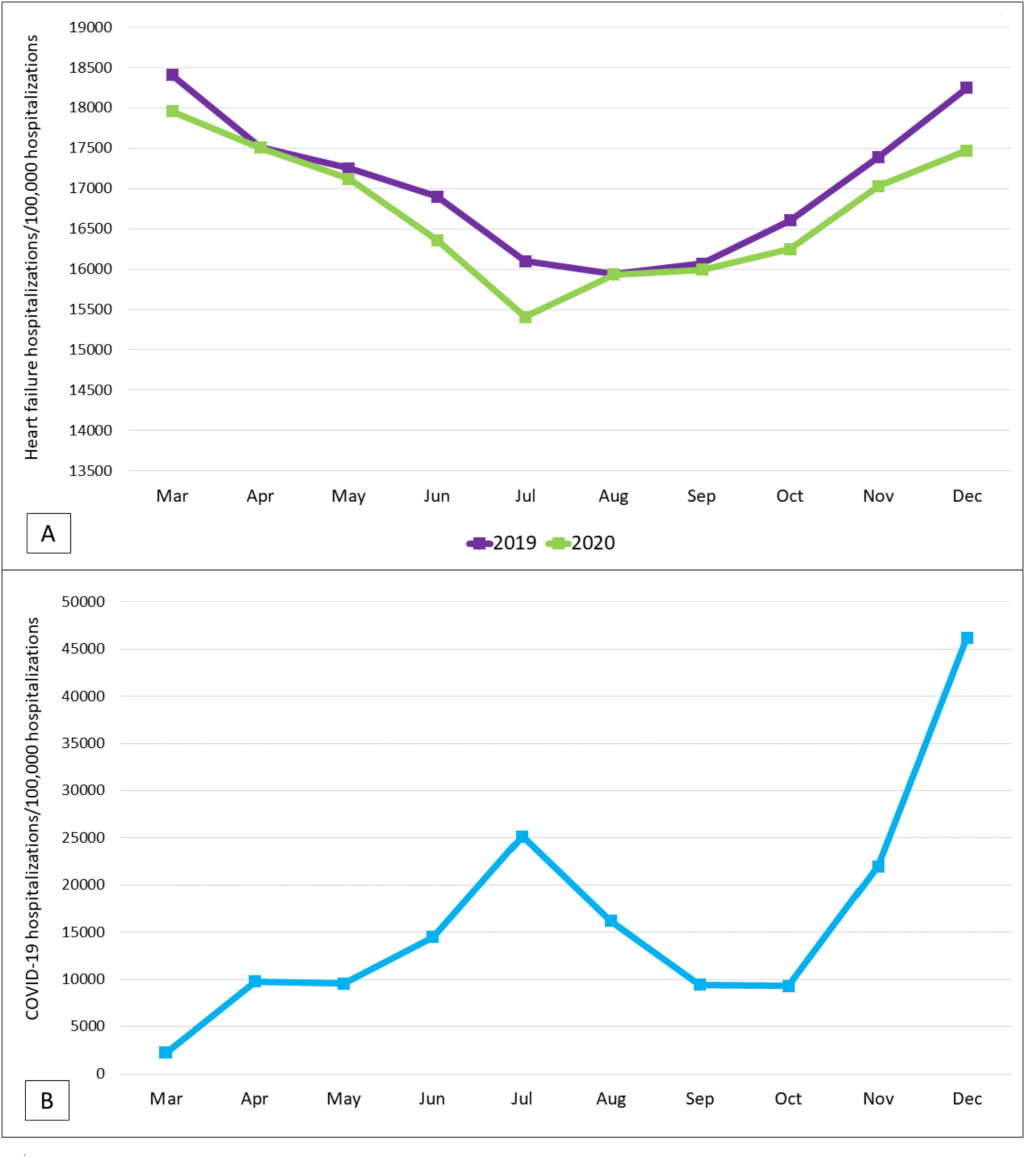
Monthly trends of **(A)** heart failure hospitalizations during March to December of 2019 and 2020; and **(B)** COVID-19 hospitalizations during March to December of 2020

Figure 1 shows the monthly trends of heart failure hospitalization rates during March to December of 2019 and March to December of 2020, compared to the monthly trends of COVID-19 hospitalization during March to December of 2020. Heart failure hospitalization rates were lower during 2020, compared to 2019. Though hospitalization rates declined during March to July across both the years, the decline was steeper during 2020. Hospitalization rates increased from July to December across both the years. However, the increases in 2020 did not catch up with the rates observed in 2019. The greatest dip in hospitalization rate observed in July 2020 coincided with the peak in COVID-19 hospitalization during the same period. However, heart failure hospitalizations increased in spite of substantial increase in COVID-19 hospitalizations during October to December of 2020.

**Fig. 2:**
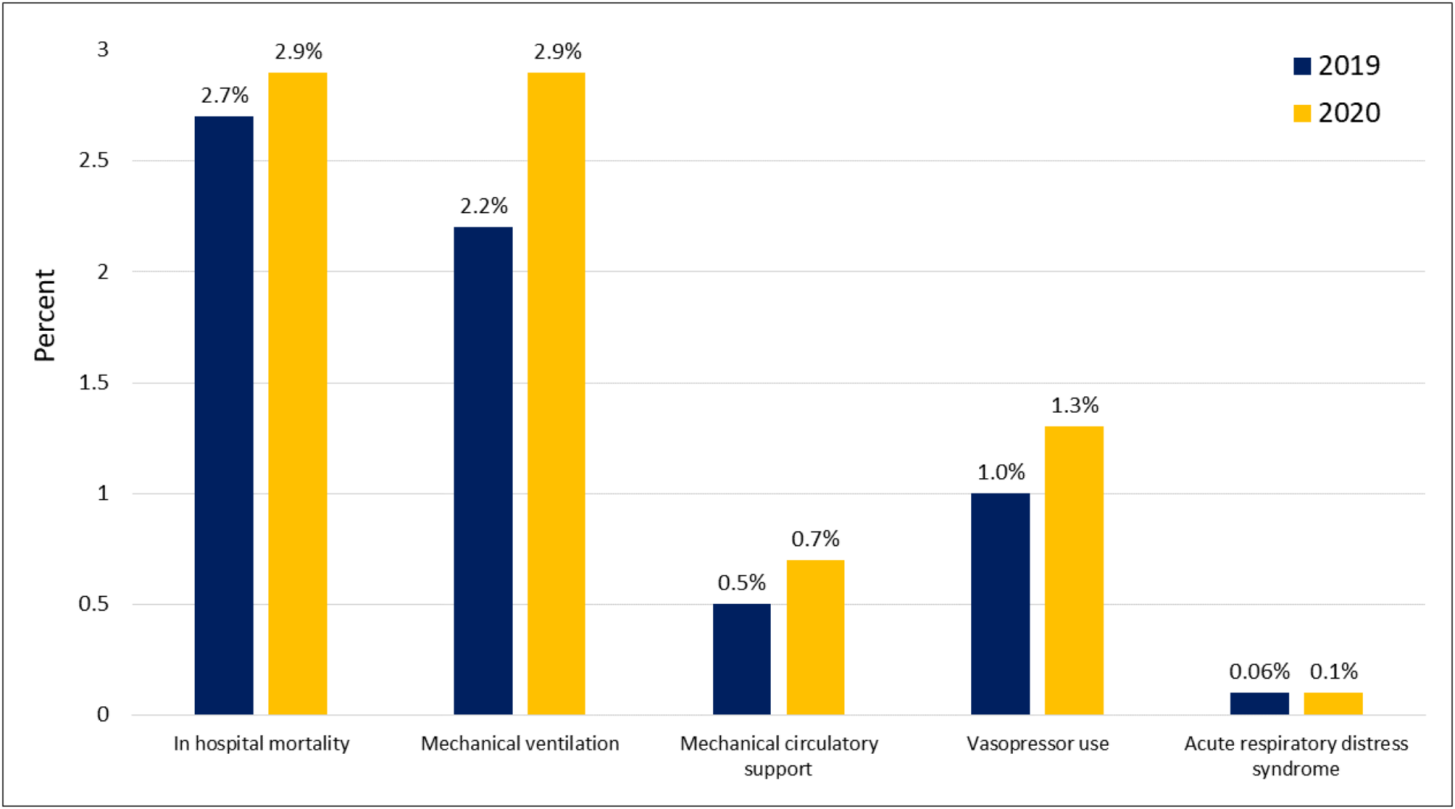
Proportion of adverse hospital outcomes among heart failure hospitalizations during March to December of 2019 and 2020

Comparison of adverse hospital outcomes across the two-time frames showed that in-hospital mortality rates were significantly higher among hospitalizations in 2020, compared to 2019 (2.9% versus 2.7%, *P* = 0.003) (Fig. 2). Similarly, mechanical ventilation (2.9% versus 2.2%, *P* < 0.001), Mechanical circulatory support (0.7% versus 0.5%. *P* < 0.001), vasopressor use (1.3% versus 1.0%, *P* < 0.001), and ARDS (0.1% versus 0.06%, *P* = 0.007) were significantly higher in 2020.

**Fig. 3:**
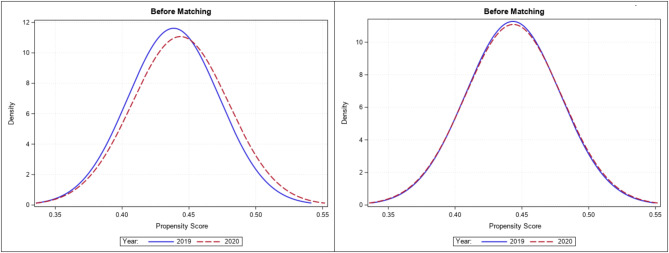
Density plots showing propensity score balance before and after matching

**Fig. 4:**
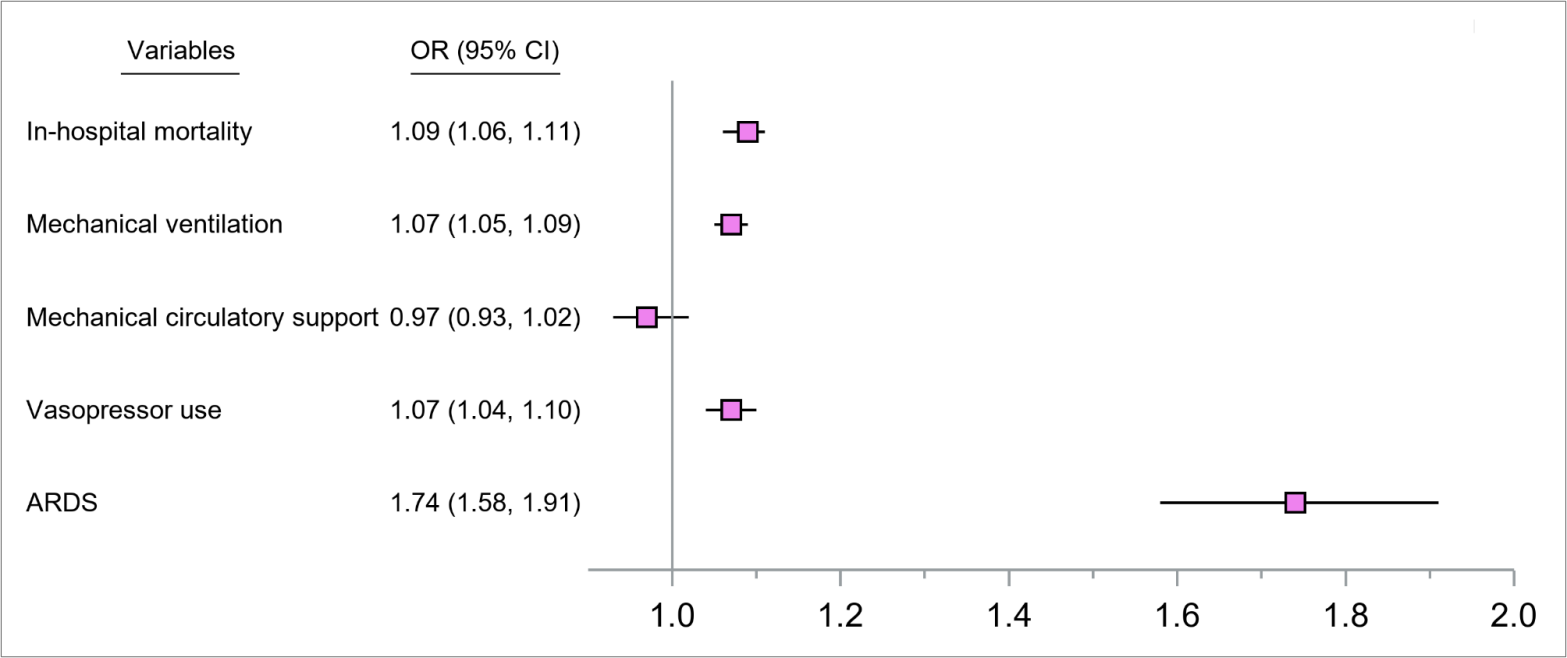
Forest plot showing odds ratios from conditional logistic regressions for the associations between hospitalization during 2019 and 2020 and outcome variables. Note: Reference is the year 2019. Covariates include age, sex, race, insurance status, and clinical risk profiles such as hypertension, diabetes mellitus, hyperlipidemia, obesity, atrial fibrillation, coagulation disorder, peripheral vascular disease, liver disease, renal failure, prior myocardial infarction (MI), prior percutaneous coronary intervention (PCI), prior coronary artery bypass graft (CABG), tobacco abuse, alcohol abuse, drug abuse, cardiac arrest, and cardiogenic shock

Comparison of density plots show that propensity score matching successfully yielded adequate covariate balance (Fig. 3).Conditional logistic regression analysis showed that the odds of adverse clinical outcomes such as in hospital mortality (OR, 1.09; 95% CI, 1.06–1.11), mechanical ventilation (OR, 1.07; 95% CI, 1.05–1.09), vasopressor use (OR, 1.07; 95% CI, 1.04–1.10), and ARDS (OR, 1.74; 95% CI, 1.58–1.91) were significantly higher among heart failure hospitalizations in 2020, compared to 2019 (Fig. 4). However, the odds of mechanical circulatory support did not differ between the two-time frames (Fig. 4).

## Discussion

In this study we looked for the impact of COVID-19 pandemic on heart failure hospitalizations in California. Trends in heart failure hospitalizations remained lower during 2020, compared to 2019. Adverse clinical outcomes such as in-hospital mortality, vasopressor use, mechanical ventilation, and ARDS were significantly higher among heart failure hospitalizations in 2020, compared to 2019.

We found that heart failure hospitalization rates were lower during 2020 and in spite of increasing trends in the latter half of the period, did not catch up with pre-COVID levels. These findings have been observed in other studies as well, which showed decreasing hospitalizations for cardiac conditions including heart failure due to COVID-19 pandemic [4, 12, 13]. A number of factors associated with COVID-19 pandemic could be responsible for these findings. Majority of the patients with heart failure could have willfully delayed treatment due to the fear of contracting COVID-19 during treatment encounters [14, 15]. In addition, the policy of diverting resources and healthcare personnel for COVID-19 management measures such as shelter-in-place and restructuring could have significantly decreased delivery of treatments and managements for non-COVID conditions such as heart failure [16, 17]. Monthly trends in hospitalization rates for heart failure showed a uniform and smooth decline during March to August of 2019 and a smooth but steeper increase during August to December of 2019. Such seasonal variations have been observed in other studies as well, which have reported increasing admissions during winter months and decreasing admissions during summer months [18–20]. Though we observed these seasonal variations in 2020 as well, the decline in hospitalization rates observed during April to July 2020 were much steeper compared to the decrease in 2019. This decline was observed subsequent to postponement of all major events and stay-at-home order issued by the government of California for the months of March and April of 2020 [21, 22]. Subsequently, after the release of a reopening plan, hospitalization rates for heart failure started to increase after a lag of 2–3 months. Nevertheless, in spite of reimposition of restrictions in the succeeding months, hospitalization rates for heart failure showed increasing trends. These increases in heart failure hospitalizations could be due to encouragements to seek care for cardiovascular conditions in spite of existing restrictions for containment of COVID-19 pandemic [23].

We observed that in-hospital mortality was significantly higher among heart failure hospitalizations in 2020, compared to 2019. Similar to our findings, a study among 1,372 heart failure patients reported that in-hospital mortality was significantly higher among patients hospitalized in 2020 (hazard ratio 2.23, *P* = 0.002). Similarly a large scale study among 101,433 patients hospitalized in 24 cardiology departments showed that in-hospital mortality rates were significantly higher among heart failure patients admitted during the pandemic [4]. A number of reasons could be responsible for these findings. It could be possible that more sicker patients with advanced stages of the disease presented for admissions during the pandemic. A previous study by Bromage at al. reported that heart failure patients admitted during 2020 were sicker and had worse New York Heart Association (NYHA) classifications, compared to the previous year, thereby resulting in higher in-hospital mortality [24]. In addition, severe acute respiratory syndrome coronavirus 2 (SARS-CoV-2) could have also directly affect the cardiovascular system and precipitated new onset heart failure leading to higher mortality rates [25]. However, the number of patients who experienced new onset heart failure after SARS-CoV-2 infection were relatively small and would not have substantially contributed to the higher mortality rates observed in 2020 [25]. Nevertheless, some studies have also reported that concomitant presence of COVID-19 and heart failure irrespective of which condition preceded one another, collectively increased the risk for mortality, especially when combined with old age and preexisting lung disease [26, 27].

We observed that vasopressor use, mechanical ventilation, and ARDS were significantly higher among heart failure hospitalizations in 2020. In the majority of COVID-19 patients, vasopressors are used to treat hypotension from sedation. It could also be used for hypotension caused by sepsis due to SARS-CoV-2 infection or concomitant bacterial infections [28, 29]. Heart failure patients with COVID-19 and experiencing hypotension are clinically managed using inotropic/vasopressor and diuretic medications [30]. However, among patients not amenable to these treatments, mechanical circulatory support is recommended as a life saving measure [30]. In our study, though we found that vasopressor use was significantly higher compared to pre-COVID-19 levels, corresponding increases in mechanical circulatory support was not observed in 2020. Therefore, we could infer that though the severity of heart failure among patients admitted in 2020 was greater than that compared to pre-COVID-19 levels, the rates of severe heart failures requiring mechanical circulatory support did not differ between the two-time frames. Increased rates of mechanical ventilation and ARDS during 2020 was not a surprising finding. Most patients with COVID-19 ARDS are managed with mechanical ventilation and evidence-based ARDS strategies [31]. In addition, mechanical ventilation could alter intrapleural and intrathoracic pressures, which could have adverse effects on cardiovascular functions such as atrial preload, ventricular afterload, heart rate, and myocardial contractility [32]. In our study, these functional deficits could also be partially responsible for greater requirements of vasopressor medications among heart failure patients who could have been mechanically ventilated.

### Limitations

Our study has some limitations. We used ICD-10 codes for identifying diagnosis and procedures, which could have led to some levels of misclassification bias due to errors in coding. The distinction between index cases and readmitted cases cannot be ascertained in SID because it deletes all personal identifiers for ensuring confidentiality of the collected data and every admission is considered an independent new admission. Consequently, our study may have overestimated hospitalization rates. SID being an administrative database, it does not have information on factors such as medication, functional classification, and left ventricular functions. We could, therefore, not ascertain the effects of these factors on our outcomes. In this study the COVID-19 data available to us was collected during 2020. There have been significant changes in understanding the pathophysiological mechanisms and management of COVID-19 since this period. Therefore, the findings in our study should be cautiously interpreted.

## Conclusion

We found that there were substantial decreases in heart failure hospitalizations due to COVID-19 pandemic in California. In spite of seasonal variations, trends in heart failure hospitalizations remained lower in 2020, compared to 2019. We also found that the rates of adverse clinical outcomes such as in-hospital mortality, vasopressor use, mechanical ventilation, and ARDS were significantly higher among patients admitted in 2020. The decrease in heart failure hospitalizations coupled with increase in adverse clinical outcomes after these hospitalizations necessitate the need to spread the message that heart failure requires prompt hospitalization and treatment irrespective of restrictive mandates during pandemics like COVID-19.

## Electronic supplementary material

Below is the link to the electronic supplementary material.

Supplementary Material 1

## Data Availability

Data is publicly available for purchase at: https://www.hcup-us.ahrq.gov/sidoverview.jsp.
