# Supplementary material for: Trends and outcomes of heart failure hospitalizations during COVID-19 pandemic
Source: BMC Public Health. 2025 Mar 4;25:864. doi: 10.1186/s12889-025-21995-y (PMC11881331; doi:10.1186/s12889-025-21995-y)
Supplement: Supplementary file 1 — Supplementary Material 1 [file 12889_2025_21995_MOESM1_ESM.doc]

Supplementary Table 1. ICD codes for hospitalizations and procedures

| **Conditions or procedures** | **ICD-10 codes** |
| --- | --- |
| COVID-19 | U071 |
| Heart failure | I110, I255, I255, I420, I501, I5020, I5021, I5022, I5023, I5030, I5031, I5032, I5033, I5040, I5041, I5042, I5043, I509, I255, I255, I420, I130, I132 |
| Tobacco use | F17, T652, Z508, Z716, Z720, P042 |
| Cardiac arrest | I46, I462, I468, I469 |
| Cardiogenic shock | R570 |
| Prior MI | I252 |
| Prior PCI | Z955 |
| Prior CABG | Z951 |
| Hyperlipidemia | E780, E781, E782, E783, E784, E785 |
| ARDS | J80 |
| Mechanical ventilation | 5A1945Z, 5A1955Z, 0BH17EZ, 0BH18EZ, 5A1935Z, 5A1945Z, 5A1955Z |
| Mechanical circulatory support | 5A15223, 5A1522F, 5A1522G,5A1522H, 5A02210, 02HA3QZ,02HA3RJ, 02HA3RZ, 02HA3RS, 02HA0RZ |
| Vasopressors | 3E030XZ, 3E033XZ, 3E040XZ, 3E043XZ, 3E050XZ, 3E053XZ, 3E060XZ, 3E063XZ |
